# Exposure to extremely low-frequency magnetic fields and childhood cancer: A systematic review and meta-analysis

**DOI:** 10.1371/journal.pone.0251628

**Published:** 2021-05-14

**Authors:** GyeongAe Seomun, Juneyoung Lee, Jinkyung Park

**Affiliations:** 1 College of Nursing, Korea University, BK21FOUR R&E Center for Learning Health Systems, Korea University, Seoul, Republic of Korea; 2 Department of Biostatistics, College of Medicine, Korea University, BK21FOUR Program in Learning Health Systems, Korea University, Seoul, Republic of Korea; 3 College of Nursing, Chonnam National University, Korea University, Seoul, Republic of Korea; Johns Hopkins University School of Medicine, UNITED STATES

## Abstract

**Background:**

Extremely low frequency magnetic fields (ELF-MFs) are classified as a possible carcinogenic factor (Group 2B). This study assessed the association between ELF-MFs and childhood cancer through a systematic review and meta-analysis.

**Methods:**

Three databases were searched in January 2020. We conducted a meta-analysis for the association between the ELF-MFs exposure level and childhood cancer.

**Results:**

A total of 33 studies were identified. Thirty studies with 186,223 participants were included in the meta-analysis. Children exposed to 0.2-, 0.3-, and 0.4-μT ELF-MFs had a 1.26 (95% confidence interval [CI] 1.06–1.49), 1.22 (95% CI 0.93–1.61), and 1.72 (95% CI 1.25–2.35) times higher odds of childhood leukemia. In childhood brain tumors, children exposed to 0.2-μT had a 0.95 (95% CI 0.59–1.56) times higher odds, and those exposed to 0.4-μT ELF-MFs had a 1.25 (95% CI 0.93–1.61). Children exposed to 0.2- and 0.4-μT ELF-MFs had a 1.10 (95% CI 0.70–1.75) and 2.01 (95% CI 0.89–4.52) times higher odds of any childhood cancers.

**Conclusions:**

Significant associations were observed between exposure to ELF-MFs and childhood leukemia. Furthermore, a possible dose-response effect was also observed.

## Introduction

The debate on the effect of electromagnetic fields (EMFs) on the human body still continues, and several studies have investigated the effect of magnetic fields that are not well shielded by objects [1–3]. The question of whether exposure to extremely low-frequency magnetic fields (ELF-MFs) from power transmission and distribution or the use of electrical appliances is associated with an increased risk of childhood cancer has engendered scientific debate [4–6]. In 2001, the ELF-MFs were classified by the International Agency for Research on Cancer (IARC) as possibly carcinogenic (Group 2B), based on the limited clinical evidence, inadequate experimental support, and the lack of plausible mechanisms at the exposure levels that were observed in epidemiological studies [7, 8]. This classification was endorsed by the subsequent weight of evidence assessments carried out by the World Health Organization (WHO) [8]. Subsequently, clinical evidence emerged from epidemiological studies on the etiology of childhood leukemia that indicated a weak association with ELF-MFs [9–12].

The WHO International Advisory Committee presented a Research Agenda for ELF-MFs whereby they indicated that epidemiological studies are of primary importance in health risk assessment, and that high-priority research needs to conduct pooled analyses of existing childhood cancer studies [13]. Until 2010, Several pooled analyses of ELF-MFs and childhood leukemia and brain tumor were conducted [4, 5, 14]. However, these studies have not conducted in accordance with the methodology of systematic review and meta-analysis. They were also pooled analyses based on only 10 studies of ELF-MFs exposure and childhood brain tumors [4], and nine and seven studies on ELF-MFs and childhood leukemia [5, 14]. Additionally, 12 studies have been conducted since those analyzes [12, 15–25]. Therefore, the pooled analyses need to be updated with the results from recent studies.

The first epidemiological study of the association between the exposure to ELF-MFs and childhood cancer was published in 1979 [26]. Over the past four decades, the potential health effects of exposure to ELF-MFs have been extensively investigated in epidemiological studies. Nonetheless, no systematic reviews and meta-analysis of the association between exposure to ELF-MFs and childhood cancer has been undertaken. Therefore, a systematic review is crucial for the scientific verification of the ELF-MFs association with cancer.

Therefore, it is necessary to comprehensively search and review the literature with an appropriate methodology, and to carry out a meta-analysis to confirm the association between exposure to ELF-MFs and childhood cancer.

### Aim

We aimed to assess the association between ELF-MFs and childhood cancer through a systematic review and meta-analysis of relevant studies.

### Design

The procedure adopted in this study followed the guidelines of the Cochrane Collaboration [27]. Moreover, the results are reported in accordance with the guidelines of the Preferred Reporting Items for Systematic Reviews and Meta-Analysis (PRISMA) [28].

## Methods

### Systematic search

Using the search strategy recommended by Cochrane [29], the search terms were chosen to obtain highly sensitive results: the keywords “electromagnetic field,” "child,” “adolescent,” and “epidemiologic studies” were combined with the Medical Subject Headings and Emtree terms S1 Table. We searched in the international databases of MEDLINE (1946 to January Week 3 2020), EMBASE (1988 to January Week 3 2020), and Web of Science (1995 to January Week 4 2020). Moreover, the reference lists of the retrieved studies were reviewed to identify additional studies that were suitable for inclusion. Literature searching has been performed by the first author who is an expert of the task with trained experiences. Our search strategy has also been confirmed by a qualified librarian.

### Study selection

Studies were searched, reviewed, and selected by two authors independently (J. P. and G. S.), using predefined criteria. Full texts for studies that met the inclusion criteria were identified and reviewed. In cases where no consensus could be reached, the issue was resolved through either further discussion or a separate examination by another author (J.L.), who is a statistical expert. However, there were no notable inconsistencies with the initial selection.

The inclusion criteria were: (1) epidemiologic studies (cohort or case-control design); (2) studies on exposure of ELF-MF; (3) studies with childhood cancer; (4) studies indicating subject number by each group exposure level; (5) based on magnetic field measurements or the calculated field; and (6) the article written in English. Abstracts presented at congresses, reviews, letters, editorials, and unpublished data were excluded.

### Quality appraisal

The Newcastle–Ottawa Scale (NOS) was used to determine the risk of bias in the studies that were included [30] in the systematic review. The NOS, a star system that allows a semi-quantitative assessment of nonrandomized study quality, contained eight items that were categorized into three major components, including selection, comparability, and exposure (case-control studies) or outcome (cohort studies). The scale ranges from zero to nine stars, where the latter represents the highest methodological quality. Two authors (J.P. and G.S.) independently assessed each study, discussed any discrepancies, and reached a consensus for all domains. In case of disagreement, the other author resolved the issue.

### Data extraction

J.P. and G.S. independently extracted the relevant data from each study, and discussed and resolved any issues that emerged. The data that were abstracted included information on: the first author, publication year, study design, country of origin, eligible population size, study population characteristics, registry group, exposure measurement, and matching variables.

### Data synthesis and analysis

We analyzed the data by using Review Manager (version 5.3) for the association between exposure to EMF and childhood cancer. Meta-analyses of the risk of childhood cancer outcomes were conducted to obtain pooled odds ratios (ORs) with the 95% confidence intervals (CIs) in a random effects model in case-control studies. The cohort studies were excluded in meta-analysis due to its differences in EMF exposure levels and outcome variables between studies. The heterogeneity among studies was estimated by using the *I*^*2*^ statistic [31, 32] as well as by the Q test. For the Q test, a *p*-value of less than 0.1 was used as an indicator of the presence of heterogeneity, the I^2^ statistic provides a measure for quantifying the heterogeneity with regard to the proportion of between-study variation based on the total variation in the estimates. In this study, an *I*^2^ value of greater than 50% was considered to denote the existence of substantial heterogeneity among the study results. We conducted analyses that were stratified by the level of exposure of EMF, and further evaluated the dose–response effect of EMF exposure. Moreover, the sensitivity analysis was performed by individually excluding each study to assess its influence of each study on the overall result of the meta-analysis.

The publication bias was examined graphically by using a funnel plot of a study’s effect size against the standard error. A two-tailed *p*-value of less than 0.05 was considered to be statistically significant.

## Results

### Search outcomes

**Fig 1:**
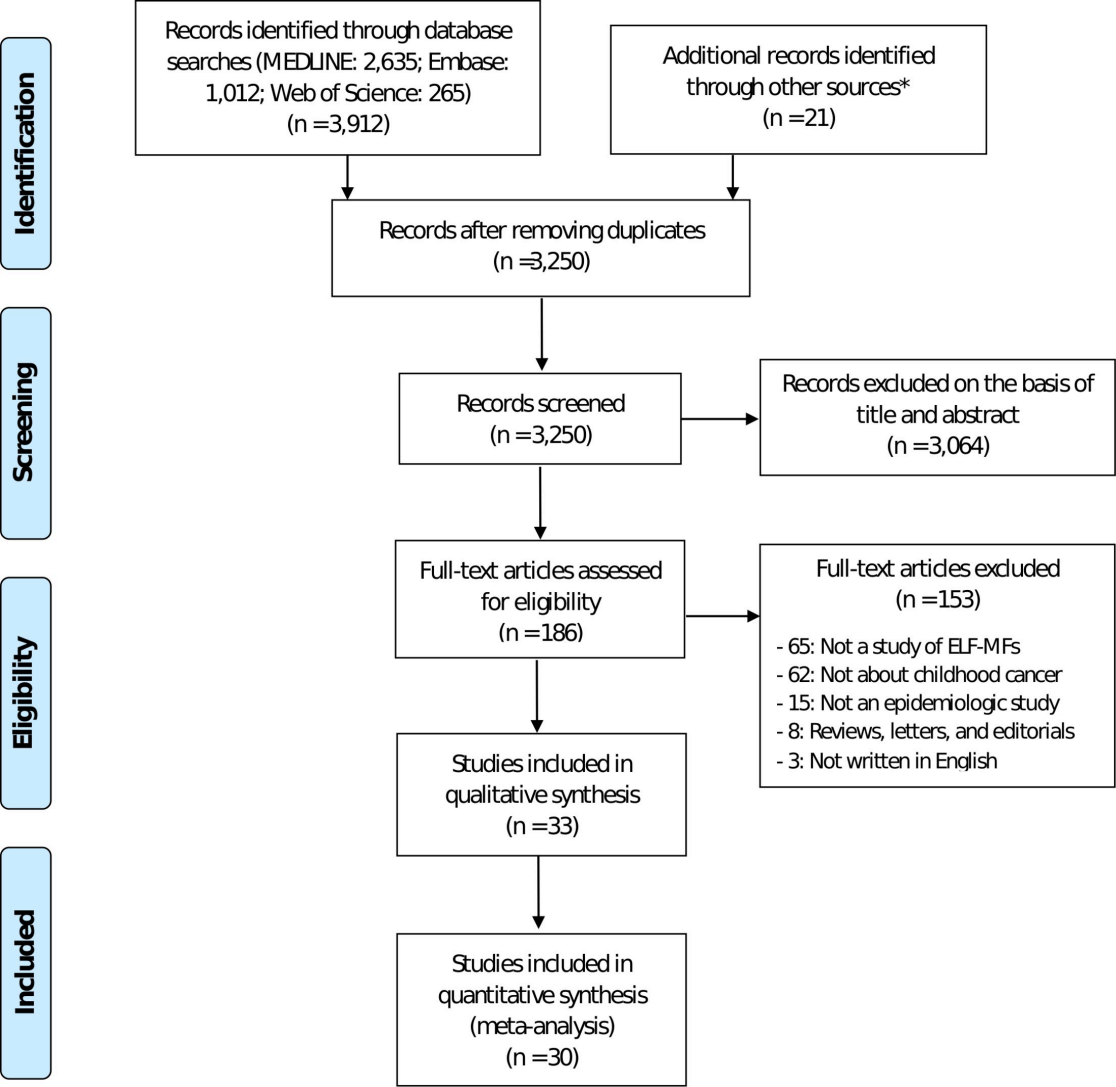
The flow chart of the search for eligible studies. PRISMA flow diagram with the process of identification, screening, eligibility, and included studies in systematic review and meta-analysis. n number of studies. * The additional studies were identified from the EMF research databases (www.emf-portal.org) and the references list of the retrieved studies.

Our systematic search found a total of 3,933 studies. After removing duplicate articles, a total of 3,250 studies were excluded for lack of eligibility for study inclusion on a review of the study title and abstract. A full-text review of the remaining 186 studies was undertaken, and a further 153 studies were excluded. Finally, 33 studies were included in the systematic review for the association of ELF-MFs and childhood cancer [9, 10, 12, 15, 18, 20, 22, 23, 33–56], and 30 studies with 186,223 were included in the meta-analysis. The selection process is summarized in Fig 1.

### Study characteristics

Table 1 presents the data extracted from all 33 studies. There were 30 case–control studies, and all except one [21] were matched case–control studies. The other three studies had a cohort design [38, 50, 57]. Published between 1988 and 2019 worldwide, all of the studies had a primary research question that was focused on the association between the exposure to ELF-MFs and childhood cancer. The age of the participants in the study population ranged from 1 day to 19 years. In 12 of the included studies, the relationship between ELF-MFs exposures with regard to any childhood cancer was researched. The cancers included lymphoma, brain tumor, and other types of cancers. With regard to the other studies, 18 articles were focused only on childhood leukemia, and some of the research was exclusively directed toward brain tumors.

**Table 1:** Characteristics of the included studies.

Study				Subjects						Exposure measurement			Matching variables		
Author	Year	Country	Study design	Cases*	Controls*	Age	Diagnosis	Year of diagnosis	Registry	Long-term measurement	Spot measurement	Calculated fields	Sex	Age	Geographic area
Savitz et al.	1988	USA	Matched case-control study	128	207	0–14 years	Any childhood cancer	1976–1983	the Colorado Central Cancer Registry		√		√	√	√
Myers et al.	1990	UK	Matched case-control study	374	588	0–14 years	Any childhood cancer	1970–1979	the Yorkshire Childhood Cancer Registry			√	√	√	√
London et al.	1991	USA	Matched case-control study	162	143	0–10 years	Childhood leukemia	1980–1987	the Los Angeles County Cancer Surveillance Program	√			√	√	
Feychting and Ahlbom	1993	Sweden	Matched case-control study	142	558	0–15 years	Any childhood cancer	1960–1985	the Swedish Cancer Registry		√	√	√	√	√
Olsen et al.	1993	Denmark	Matched case-control study	1,707	4,788	0–15 years	Any childhood cancer	1968–1986	The nationwide cancer registration system			√	√	√	
Verkasalo et al.^a^	1993	Finland	cohort study	29	1027	0–19 years	Any childhood cancer	1970–1989				√			
Preston-Martin et al.	1996	USA	Matched case-control study	437	433	0–19 years	Childhood brain tumor	1984–1991	the Los Angeles portion of a multicenter	√			√	√	√
Linet et al.	1997	USA	Matched case-control study	629	619	0–14 years	Childhood lymphocytic leukemia	1989–1994	the Children’s Cancer Group	√			√	√	√
Michaelis et al.	1997	Germany	Matched case-control study	176	414	0–14 years	Any childhood cancer	1991–1994	the German Childhood Cancer Registry	√			√	√	√
Tynes et al.	1997	Norway	Matched case-control study	532	2,112	0–14 years	Any childhood cancer	1965–1989	the Cancer Registry of Norway	√		√	√	√	
Dockerty et al.	1998	New Zealand	Matched case-control study	303	303	0–14 years	Childhood leukemia, brain tumor, other solid cancers	1990–1993	the New Zealand Cancer Registry	√			√	√	
Green et al.	1999	Canada	Matched case-control study	189	381	0–14 years	Childhood leukemia	1985–1993	the Pediatric Oncology Group Registry	√			√	√	√
McBride et al.	1999	Canada	Matched case-control study	399	399	0–10 years	Childhood lymphatic leukemia	1990–1995	In British Colombia and Quebec	√			√	√	√
UKCCS investigators	1999	UK	Matched case-control study	2226	2226	0–14 years	Any childhood cancer	1991–1996	the UKCCS	√			√	√	√
Kleinerman et al.	2000	USA	Matched case-control study	408	408	0–14 years	Childhood lymphoblastic leukemia	1989–1993	the Children’s Cancer Group			√	√	√	√
Bianchi et al.	2000	Italy	Matched case-control study	101	412	0–14 years	Childhood leukemia	1976–1992	the National Electricity Board		√	√	√	√	√
Schüz et al.	2001	Germany	Matched case-control study	514	1,301	0–14 years	Childhood leukemia	1992–1994	the German Childhood Cancer Registry (GCCR)	√			√	√	√
Foliart et al.	2006	USA	cohort study			1–15 years	Childhood lymphoblastic leukemia	1996–2004	the Pediatric Oncology Group(POG)	√					
Kabuto et al.	2006	Japan	Matched case-control study	312	603	0–15 years	Childhood leukemia	1999–2001	the 5 major children’s cancer study groups in Japan	√			√	√	√
Feizi and Arabi	2007	Iran, Tabriz	Matched case-control study			0–14 years	Childhood leukemia	1998–2004	the Children’s Hospital of Tabriz			√	√	√	√
Svendsen et al.	2007	Germany	cohort study			1–14 years	Childhood lymphoblastic leukemia	1988–1994		√					
Kroll et al.	2010	UK	Matched case-control study	9695	9695	0–14 years	Any childhood cancer	1962–1995	the National Registry of Childhood Tumours			√	√	√	√
Malagoli et al.	2010	Italy	Matched case-control study	46	184	0–13 years	Childhood leukemia	1986–2007	the Associazione Italiana Ematologia Oncologia Pediatrica (AIEOP)			√	√	√	√
Saito et al.	2010	Japan	Matched case-control study	55	99	0–14 years	Childhood brain tumor	1999–2002	network of 107 hospitals	√	√		√	√	√
Does et al.	2011	USA	Matched case-control study			0–8 years	Any childhood cancer	2002–2007	the Northern and Central California (NCCLS)		√		√	√	√
Wünsch Filho et al.	2011	Brazil	Matched case-control study	162	565	0–18 years	Childhood lymphocytic leukemia	2003–2009	Eight hospitals in the State of Sao Paulo	√			√	√	√
Jirik et al.	2012	Czech Republic	Matched case-control study	79	79	0–14 years	Childhood leukemia	-		√			√	√	√
Ba Hakim et al.	2014	Malaysia	case-control study	108	118	0–14 years	Childhood leukemia	2001–2007	University Kebangsaan Malaysia Hospital and Kuala Lumpur General Hospital			√			
Bunch et al.	2015	UK	Matched case-control study	52,525	52,525	0–14 years	Any childhood cancer	1962–2008	the national registry of childhood tumours (NRCT)			√	√	√	√
Pedersen et al.	2015	Denmark	Matched case-control study	3,277	9,129	0–14 years	Childhood leukemia, lymphoma, brain tumor	1968–2003	the Danish Data Protection Agency			√	√	√	√
Salvan et al.	2015	Italy	Matched case-control study	412	587	0–10 years	Childhood leukemia	1998–2001	the SETIL case-control study	√			√	√	√
Kheifets et al.	2017	USA	Matched case-control study	5,788	5,788	0–15 years	Childhood leukemia	1986–2008	the California Cancer Registry			√	√	√	
Crespi et al.	2019	USA	Matched case-control study	4,879	4,835	0–14 years	Childhood leukemia	1986–2008	the California Cancer Registry			√	√	√	√

a: case control data generated from the original cohort by original author

*: Numbers of cases and controls presented in the table are for subjects with available measurements

The included three cohort studies were unable to perform meta-analysis due to differences in exposure levels and outcomes between studies. In a cohort study investigating the risk of cancer in children living close to overhead power lines with a magnetic field of 0.01-uT, the standardized incidence ratio was 0.97 (95% confidence interval [CI], 0.8-1.l), with no statistically significant increases in all cancers [38]. Two of the three cohort studies examined the association between ELF-MFs exposure and survival after diagnosis of childhood leukemia [50, 57]. The results were a survival risk (Hazard Ratio, HR) of 1.9 (95% CI, 0.8–4.9) at 0.3-μT [57] and 3.0 (95% CI, 0.9–9.8) at 0.2-μT [50].

The study characteristics included the year of diagnosis and the registry group (Table 1). The methods for the EMFs exposure assessments are presented from long-term measurements, spot measurements, and the calculated field. For 12 studies, the EMF exposure was calculated and presented through a measurement of the distance to the high-voltage line. In the included case–control studies, the cases and controls were matched by the sex, age, and geographic areas, and the confounding factors were adjusted. In five studies [10, 35, 37, 41, 58], although they did not match with the geographic area variables, we attempted to control for the factors that influenced the outcome, such as selecting a control group from each of the study’s registries.

The absolute numbers of childhood cancer cases and controls for the exposure level of the ELF-MFs were presented in Table 2 along with the OR and 95% CI. The level of exposure to ELF-MFs were predominantly 0.2, 0.3, and 0.4-μT, and the results that were derived from most of the studies were compared with 0.1-μT or less exposure.

**Table 2:** Absolute numbers of childhood cancer cases and controls by study and exposure level.

Author	Year	Cases	Controls	Electro-magnetic Field Level	OR	95%CI
***Leukemia***						
Savitz et al.	1988	5	16	≥0.2 μT	1.41	0.57–3.50
		31	191	< 0.2 μT	1.0	
London et al.	1991	20	11	≥0.27 μT	1.48	0.66–3.29
		24	22	0.12-< 0.27 μT	0.89	0.43–1.71
		35	42	0.01-<0.12 μT	0.68	0.39–1.17
		85	69	< 0.01 μT	1.0	
Feychting and Ahlbom	1993	4	70	≥0.2 μT		
		1	67	0.1-<0.2 μT		
		19	207	<0.1 μT		
Olsen et al.	1993	3	1	≥0.4 μT	6.0	0.8–44
		1	7	0.1-<0.4 μT	0.3	0.0–2.0
		829	1658	Not exposed		
Verkasalo et al.	1993	1	7	≥0.4 μT	6.21	0.68–56.9
		1	10	0.2-<0.4 μT	4.11	0.48–35.1
		0	19	0.1-<0.2 μT	-	
		27	991	<0.1 μT		
Linet et al.	1997	5	5	≥0.5 μT	1.01	0.26–3.99
		10	2	0.4-<0.5 μT	6.41	1.30–31.73
		14	11	0.3-<0.4 μT	1.46	0.61–3.50
		29	26	0.2-<0.3 μT	1.31	0.68–2.51
		107	106	0.1-<0.2 μT	1.15	0.79–1.65
		298	313	<0.1 μT		
Michaelis et al.	1997	9	8	≥0.2 μT	2.3	0.8–6.7
		165	406	< 0.2 μT		
Tynes et al.	1997	1	14	≥0.14 μT	0.3	0.0–2.1
		8	19	0.05-<0.14 μT	1.8	0.7–4.2
		139	546	<0.05 μT		
Dockerty et al.	1998	5	1	≥0.2 μT	15.5	1.1–224
		4	5	0.1-<0.2 μT	1.4	0.3–7.6
		31	34	<0.1 μT		
Green et al.	1998	21	46	≥0.13 μT	1.68	0.58–4.82
		27	41	0.07-<0.13 μT	1.44	0.50–4.16
		19	49	0.03-<0.07 μT	1.30	0.47–3.55
		15	44	<0.03 μT		
McBride et al.	1999	8	6	≥0.5 μT	0.89	0.24–3.36
		5	8	0.4-<0.5 μT	0.44	0.11–1.80
		11	9	0.3-<0.4 μT	1.24	0.47–3.26
		30	29	0.2-<0.3 μT	1.06	0.57–1.99
		63	95	0.1-<0.2 μT	0.70	0.46–1.06
		176	192	<0.1 μT		
UKCCS investigators	1999	5	3	≥0.4 μT	1.68	0.40–7.10
		16	20	0.2-<0.4 μT	0.78	0.40–1.52
		57	73	0.1-<0.2 μT	0.78	0.55–1.12
		995	977	<0.1 μT		
Kleinerman et al.	2000	35	35	≥1.3 μT	0.98	0.59–1.63
		26	36	0.66-<1.30 μT	0.72	0.41–1.27
		47	34	0-<0.66 μT	1.30	0.80–2.21
		297	300	Not exposed		
Bianchi et al.	2000	3	3	≥0.1 μT	4.51	0.88–23.17
		6	8	0.001-<0.1 μT	3.29	1.11–9.73
		92	401	Not exposed		
Schüz et al.	2001	3	3	≥0.4 μT	5.81	0.78–43.2
		6	15	0.2-<0.4 μT	1.16	0.43–3.11
		33	73	0.1-<0.2 μT	1.15	0.73–1.81
		472	1210	<0.1 μT		
Kabuto et al.	2006	6	5	≥0.4 μT	2.56	0.76–8.58
		12	20	0.2-<0.4 μT	1.12	0.53–2.36
		18	36	0.1-<0.2 μT	0.91	0.50–1.63
		276	542	<0.1 μT		
Feizi and Arabi	2007	15	5	≥0.45 μT	3.60	1.11–12.39
		45	54	< 0.45 μT		
Kroll et al.	2010	2	1	≥0.4 μT	2.00	0.18–22.04
		0	2	0.2-<0.4 μT	-	
		6	3	0.1-<0.2 μT	2.00	0.50–7.99
		9645	9647	<0.1 μT		
Malagoli et al.	2010	1	2	≥0.4 μT	2.1	0.2–26.2
		2	3	≥0.1 μT	6.7	0.6–78.3
		27	129	<0.1 μT		
Does et al.	2011	3	6	≥0.3 μT	0.57	0.14–2.36
		5	6	0.2-<0.3 μT	1.03	0.30–3.55
		22	12	0.1-<0.2 μT	1.98	0.94–4.17
		215	245	<0.1 μT		
Wünsch Filho et al.	2011	11	34	≥0.3 μT	1.09	0.33–3.61
		38	137	0.1-<0.3 μT	0.75	0.36–1.55
		113	394	<0.1 μT		
Jirik et al.	2012	31	32	≥0.2 μT	0.93	0.45–1.93
		48	47	< 0.2 μT		
		13	14	≥0.4 μT	0.90	0.37–2.22
		66	65	< 0.4 μT		
Ba Hakim et al.	2014	11	10	≥0.3 μT	0.82	0.33–2.00
		97	108	< 0.3 μT		
Bunch et al.	2015	2	1	≥0.4 μT	2.00	0.18–22.06
		3	4	0.2-<0.4 μT	0.92	0.20–4.17
		1	2	0.1-<0.2 μT	0.80	0.07–9.10
		17304	20952	<0.1 μT		
Pedersen et al.	2015	5	6	≥0.4 μT	1.67	0.51–5.46
		5	13	0.1-<0.4 μT	0.77	0.27–2.16
		1526	3053	<0.1 μT		
Salvan et al.	2015	15	24	≥0.3 μT	0.75	0.38–1.50
		20	13	0.2-<0.3 μT	2.24	1.03–4.88
		39	69	0.1-<0.2 μT	0.81	0.53–1.25
		335	463	<0.1 μT		
Kheifets et al.	2017	17	11	≥0.4 μT	1.48	0.69–3.19
		14	15	0.2-<0.4 μT	0.97	0.46–2.02
		24	27	0.1-<0.2 μT	0.84	0.48–1.46
		5733	5735	<0.1 μT		
Crespi et al.	2019	17	11	≥0.4 μT	1.50	0.70–3.23
		14	15	0.2-<0.4 μT	0.97	0.47–2.02
		24	27	0.1-<0.2 μT	0.84	0.48–1.47
		4824	4782	<0.1 μT		
***Lymphomas***						
Savitz et al.	1988	2	16	≥0.2 μT	1.81	0.48–6.88
		11	191	< 0.2 μT		
Olsen et al.	1993	1	1	≥0.4 μT	5.0	0.3–82
		2	2	0.1-<0.4 μT	5.0	0.7–36
		247	1247	Not exposed		
Tynes et al.	1997	2	3	≥0.14 μT	2.5	0.4–15.5
		1	5	0.05-<0.14 μT	1.0	0.1–8.7
		27	117	<0.0 μT		
Pedersen et al.	2015	2	4	≥0.4 μT	2.50	0.46–13.65
		3	12	0.1-<0.4 μT	1.25	0.35–4.43
		412	2069	<0.1 μT		
***Brain tumor (Central Nervous System)***						
Savitz et al.	1988	2	16	≥0.2 μT	0.82	0.23–2.93
		23	191	< 0.2 μT		
Feychting and Ahlbom	1993	5	70	≥0.2 μT		
		8	67	0.1-<0.2 μT		
		10	207	<0.1 μT		
Olsen et al.	1993	2	1	≥0.4 μT	6.0	0.7–44
		1	8	0.1-<0.4 μT	0.4	0.1–2.8
		621	1863	Not exposed		
Preston-Martin et al.	1996	11	11	≥0.2 μT	1.2	0.4–3.2
		19	12	0.1-<0.2 μT	1.8	0.7–4.5
		29	22	0.05-<0.1 μT	1.5	0.7–3.2
		47	54	<0.05 μT		
Tynes et al.	1997	4	23	≥0.14 μT	0.7	0.7–2.1
		8	17	0.05-<0.14 μT	1.9	0.8–4.6
		144	599	<0.0 μT		
UKCCS investigators	1999	0	2	≥0.4 μT		
		3	4	0.2-<0.4 μT	0.70	0.16–3.17
		25	10	0.1-<0.2 μT	2.44	1.17–5.11
		359	371	<0.1 μT		
Kroll et al.	2010	1	3	≥0.4 μT	0.33	0.0.-3.20
		1	0	0.2-<0.4 μT	-	
		2	4	0.1-<0.2 μT	0.50	0.09–2.73
		6580	6577	<0.1 μT		
Saito et al.	2010	3	1	≥0.4 μT	10.9	1.05–113
		2	4	0.2-<0.4 μT	1.58	0.25–9.83
		3	8	0.1-<0.2 μT	0.74	0.17–3.18
		47	86	<0.1 μT		
Bunch et al.	2015	0	1	≥0.4 μT	-	
		0	0	0.2-<0.4 μT	-	
		4	1	0.1-<0.2 μT	4.65	0.51–42.23
		12294	15258	<0.1 μT		
Pedersen et al.	2015	4	9	≥0.4 μT	1.33	0.41–4.33
		8	23	0.1-<0.4 μT	1.04	0.46–2.36
		1312	3940	<0.1 μT		
***Any Childhood cancer***						
Savitz et al.	1988	13	16	≥0.2 μT	1.35	0.63–2.90
		115	191	< 0.2 μT		
Myers et al	1990	1	4	≥0.1 μT	0.39	0.04–4.09
		15	17	0.01-<0.1 μT	1.35	0.61–3.01
		358	567	<0.01 μT		
Feychting and Ahlbom	1993	16	70	≥0.2 μT	0.9	0.5–1.7
		20	67	0.1-<0.2 μT	1.2	0.7–2.1
		53	207	<0.1 μT		
Olsen et al.	1993	6	3	≥0.4 μT	5.6	1.6–19
		4	17	0.1-<0.4 μT	0.7	0.2–2.0
		4	21	<0.1 μT	0.6	0.2–17
		1677	4698	Not exposed		
Verkasalo et al.	1993	1	7	≥0.4 μT		
		1	10	0.2-<0.4 μT		
		0	19	0.1-<0.2 μT		
		25	991	<0.1 μT		
Tynes et al.	1997	12	51	≥0.14 μT	0.9	0.5–1.8
		24	51	0.05-<0.14 μT	1.9	1.2–3.3
		464	1902	<0.0 μT		
UKCCS investigators	1999	8	9	≥0.4 μT	0.89	0.34–2.29
		31	35	0.2-<0.4 μT	0.87	0.53–1.42
		120	128	0.1-<0.2 μT	0.93	0.72–1.19
		2067	2054	<0.1 μT		
Pedersen et al.	2015	11	19	≥0.4 μT	1.63	0.77–3.46
		16	48	0.1-<0.4 μT	0.98	0.55–1.74
		3250	9062	<0.1 μT		

OR = Odds ratio; CI = confidence interval

### Risk of bias assessment

The risk of bias in the studies was evaluated by the NOS for all 30 articles included in the meta-analysis. A total of 30 articles that met all of the selection criteria were assessed for their quality. The results associated with the quality ratings of the retrieved studies are shown in Table 3. A total score of 7 and more indicated high-quality studies.

**Table 3:** Newcastle-Ottawa Scale assessment of the quality for the included studies.

Author	Year	SELECTION				COMPARABILITY	EXPOSURE			Total Scores
		Case Definition	Representativeness of the Cases	Selection of Controls	Definition of Controls	Comparability	Ascertainment of Exposure	Same method of ascertainment	Non-Response Rate	
Savitz et al.	1988	✵	✵	✵	✵	✵✵	✵	✵		8
Myers et al.	1990	✵	✵	✵	✵	✵✵	✵	✵		8
London et al.	1991	✵	✵	✵	✵	✵	✵	✵		7
Feychting and Ahlbom	1993	✵	✵	✵	✵	✵✵	✵	✵		8
Olsen et al.	1993	✵	✵	✵	✵	✵	✵	✵		7
Verkasalo et al.^a^	1993	✵	✵	✵	✵	✵	✵	✵		7
Preston-Martin et al.	1996	✵	✵	✵	✵	✵✵	✵	✵		8
Linet et al.	1997	✵	✵	✵	✵	✵✵	✵	✵		8
Michaelis et al.	1997	✵	✵	✵	✵	✵✵	✵	✵		8
Tynes et al.	1997	✵	✵	✵	✵	✵	✵	✵		7
Dockerty et al.	1998	✵	✵	✵	✵	✵	✵	✵		7
Green et al.	1999	✵	✵	✵	✵	✵✵	✵	✵		8
McBride et al.	1999	✵	✵	✵	✵	✵✵	✵	✵		8
UKCCS investigators	1999	✵	✵	✵	✵	✵✵	✵✵	✵		9
Kleinerman et al.	2000	✵	✵	✵	✵	✵✵	✵	✵		8
Bianchi et al.	2000	✵	✵	✵	✵	✵✵	✵	✵		8
Schüz et al.	2001	✵	✵	✵	✵	✵✵	✵	✵		8
Kabuto et al.	2006	✵	✵	✵	✵	✵✵	✵	✵		8
Feizi and Arabi	2007	✵	✵	✵	✵	✵✵	✵	✵		8
Kroll et al.	2010	✵	✵	✵	✵	✵✵	✵	✵		8
Malagoli et al.	2010	✵	✵	✵	✵	✵✵	✵	✵		8
Saito et al.	2010	✵	✵	✵	✵	✵✵	✵	✵		8
Does et al.	2011	✵	✵	✵	✵	✵	✵	✵		7
Wünsch Filho et al.	2011	✵	✵	✵	✵	✵✵	✵	✵		8
Jirik et al.	2012	✵	✵	✵	✵	✵✵	✵	✵		8
Ba Hakim et al.	2014	✵	✵	✵	✵	✵	✵	✵		7
Bunch et al.	2015	✵	✵	✵	✵	✵✵	✵	✵		8
Pedersen et al.	2015	✵	✵	✵	✵	✵✵	✵	✵		8
Salvan et al.	2015	✵	✵	✵	✵	✵✵	✵	✵		8
Kheifets et al.	2017	✵	✵	✵	✵	✵	✵	✵		7
Crespi et al.	2019	✵	✵	✵	✵	✵✵	✵	✵		8

✵ denotes 1 point. The empty cells indicate that the study did not have any points for that category

### Meta-analysis

We identified 30 studies with 186,233 subjects that report results on the association between ELF-MFs and childhood cancer. Meta-analyses were conducted for pediatric leukemia, brain tumors, and any childhood cancers by level of exposure to ELF-MFS.

#### Childhood leukemia

**Fig 2:**
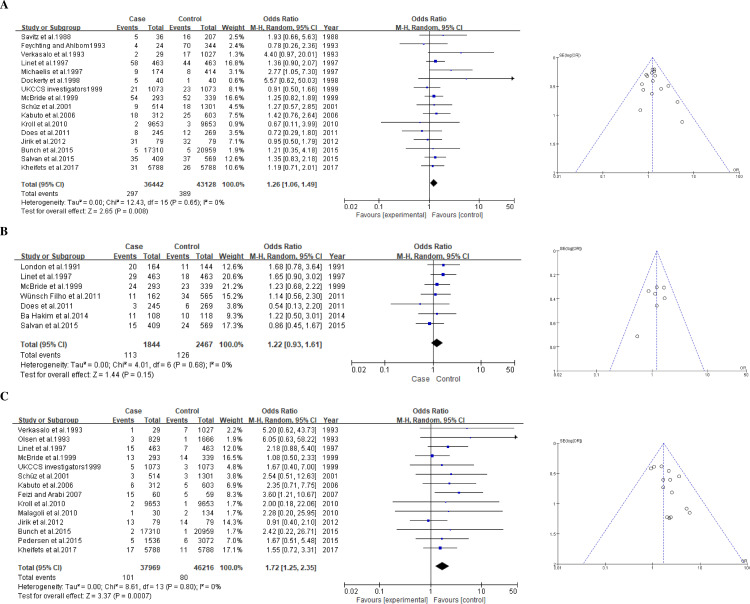
Forest plot and funnel plot of exposure to ELF-MFs and risk of childhood leukemia. The pooled odds ratio of childhood leukemia by each exposure level of ELF-MFs: **A** 0.2-μT exposure level; **B** 0.3-μT exposure level; **C** 0.4-μT exposure level.

A total of 27 studies including 45,029 cases and 55,376 controls were included in the meta-analysis for leukemia (Table 2). For a group exposed to 0.2-μT and higher level of ELF-MFs, the association with childhood leukemia was significant (pooled summary OR, 1.26; 95% CI, 1.06–1.49) in a random effect model (Fig 2A) without any significant heterogeneity among studies being shown (I^2^ = 0%). For the 0.3-μT exposure level of ELF-MFs, the pooled summary OR was 1.22 (0.93–1.61) without any significant heterogeneity (I^2^ = 0%; Fig 2B). For a 0.4-μT exposure level of ELF-MFs, the pooled summary OR was 1.72 (1.25–2.35; Fig 2C).

We checked for the presence of publication bias through a funnel plot and found that there was no evidence of publication bias (Fig 2). With the results shown in the meta-analysis that indicate the risk of childhood leukemia by the exposure level of ELF-MFs, the odds level increased as the exposure level increased (1.26→1.22→1.72), which shows a dose-response effect. This confirms an association between exposure to ELF-MFs and a high risk of childhood leukemia.

#### Pediatric brain tumor

**Fig 3:**
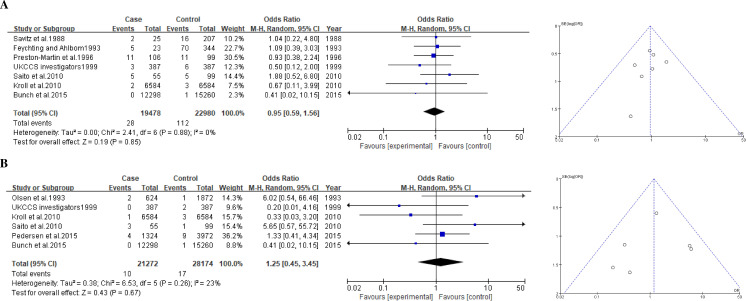
Forest plot and funnel plot of exposure to ELF-MFs and risk of childhood brain tumor. The pooled odds ratio of childhood brain tumor by each exposure level of ELF-MFs: **A** 0.2-μT exposure level; **B** 0.4-μT exposure level.

A total of 10 studies including 21,582 cases and 29,463 controls were included in the meta-analysis of the association between exposure to ELF-MFs and pediatric brain tumors (Table 2). In this study, the meta-analysis was performed by the exposure levels of 0.2 and 0.4-μT. The pooled summary OR for the 0.2-μT exposure level of ELF-MFs was 0.95 (0.59–1.56) in a random effect model, and no significant heterogeneity was detected (*I*^2^ = 0%; Fig 3A). This non-significant association was observed for the 0.4-μT exposure level (1.25, 0.93–1.61), and the heterogeneity might not be important (*I*^2^ = 23%; Fig 3B). We confirmed through the funnel plot that there was no indication of publication bias for the studies included in our meta-analysis (Fig 3).

#### Any childhood cancers

**Fig 4:**
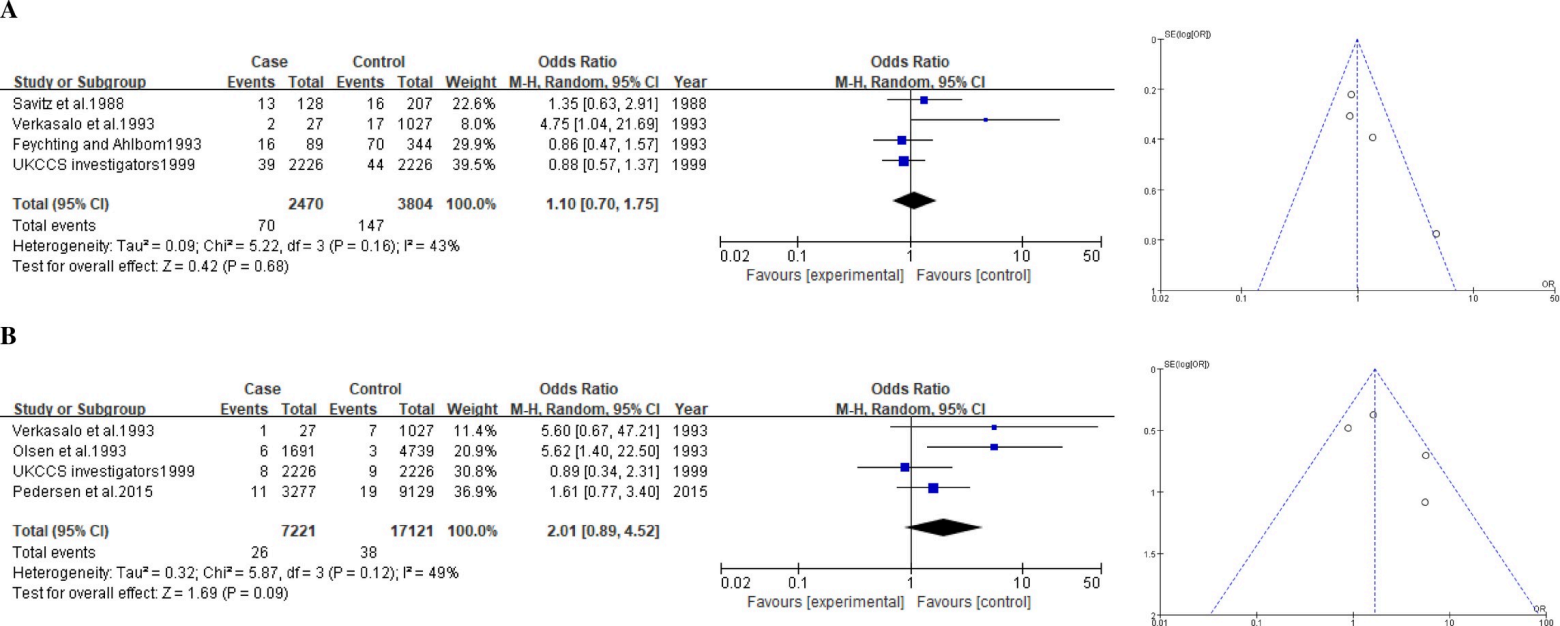
Forest plot and funnel plot of exposure to ELF-MFs and risk of any childhood cancer. The pooled odds ratio of any childhood cancer by each exposure level of ELF-MFs: **A** 0.2-μT exposure level; **B** 0.4-μT exposure level.

The results of six study were used to examine an association of exposure to ELF-MFs with any childhood cancers. The pooled summary OR of the 0.2-μT exposure level of ELF-MFs was 1.10 (0.70–1.75) (Fig 4A), whereas that for the 0.4-μT exposure level was 2.10 (0.70–1.75) (Fig 4B). The size of significant heterogeneity was moderate (*I*^2^ = 43–49%), and there was evidence of publication bias (Fig 4).

#### Sensitivity analysis

The sensitivity analysis was performed after excluding either studies that used calculated fields measurement method or studies that identified very small cases even though many participants were examined and, hence, affected to pooled estimate of meta-analysis significantly [15, 22]. The pooled summary OR for childhood leukemia with 11 studies that directly measured the level of exposure was 1.27 (1.05–1.53) at exposure levels above 0.2-μT, and 1.44 (0.95–2.17) at exposure levels above 0.4-μT in 6 studies. Excluding 2 studies with very small cases [15, 22], the pooled summary OR of 12 studies was 1.27 (1.07–1.51) at above 0.2-μT, and 1.70 (1.24–2.34) at above 0.4-μT.

## Discussion

We conducted a meta-analysis on each type of cancer to derive the large-scale integrated values for the association between ELF-MFs exposure and childhood cancer among more than 85,000 children diagnosed with childhood cancer in 15 countries. In this study, based on the systematic review methodology, all articles that presented pediatric cancer, including leukemia, lymphoma, and brain tumor, were included in a comprehensive search. Pooled analysis studies have been conducted on the association between childhood cancer and ELF-MFs exposure [4, 5, 14, 59]. However, such studies did not include articles that were identified through a comprehensive search, and pooled analyses were performed by using a modified level of exposure to ELF-MFs. In this regard, this study is the first to conduct a systematic review of the association between ELF-MFs exposure and childhood cancer, and to have rigorously performed a meta-analysis. Additionally, while previous pooled analyses had been performed with 2 to 10 studies, this study expands the results using up to date 30 studies.

The existence of between-study heterogeneity can generate a statistical problem when attempting to interpret the results of the meta-analysis. Ideally, the studies, whose results are being combined in the meta-analysis, should all be undertaken in the same way and with the same experimental protocols: study heterogeneity is a term used to indicate that this ideal is not fully met [27]. Meta-analysis is a method for combining the results of different studies to obtain a quantified synthesis, and it increases the power of statistical analyses by pooling the results of all available studies. A meaningful explanation is possible when attempting to estimate the combined effect of a similar study group by using meta-analysis, and the presence of heterogeneity can affect the statistical validity of the estimated summary of that effect [60]. The results of the present study are meaningful because the meta-analysis was conducted for the type of childhood cancer that occurred by the exposure levels of ELF-MF–that is, integrated into the random effects model because of potential unexplained heterogeneity [27].

This study showed that children exposed to 0.2-μT ELF-MFs had a 1.26 (95% CI 1.06–1.49) times higher odds of child leukemia, those exposed to 0.3-μT had a 1.22 (0.93–1.61) times higher odds, and those exposed to 0.4-μT had a 1.72 (1.25–2.35) times higher odds. These results are based on statistical homogeneity (I^2^ = 0%) as well as an absence of any evidence for publication bias. In addition, this study derived a large-scale integrated value using larger number of included studies than previous studies. Our results showed slightly higher pooled estimates than previous results, which are 1.07–1.08, 1.11–1.16, and 1.44–2.00 times higher odds for exposures to 0.2-, 0.3-, and 0.4-μT, respectively [5, 14]. Furthermore, the higher degree of intensity of the association between the exposure to ELF-MFs and childhood leukemia than previous studies was obtained because we observed the dose–response effects of an increase in the pooled OR value with increasing exposure level to ELF-MFs was observed.

According to the pooled OR of 10 studies with 7,247 subjects, children exposed to 0.2-μT ELF-MFs had a 0.95 (0.59–1.56) times higher odds of childhood brain tumors, and children exposed to 0.4-μT ELF-MFs had a 1.25 (0.93–1.61) times higher odds. However, the estimates were not statistically significant. Based on previous pooled analysis, studies showed a 0.95 times on exposure to 0.2-μT and 1.14-times higher odds on exposure to 0.4-μT ELF-MFs, and similar results were found in this study [4].

According to the pooled OR value of four studies, children exposed to 0.2-μT ELF-MFs had a 1.10 (0.70–1.75), and those exposed to 0.4-μT ELF-MFs had a 2.01 (0.89–4.52) times higher odds of any childhood cancer, However, it did not show a high-intensity relation. There have been no pooled analysis studies of any childhood cancer conducted thus far.

Public concern about EMFs is considerably high, and many countries have implemented a lot of policies related to EMFs [61, 62]. Any change in the epidemiological evidence for childhood cancer would be important to these public policies besides the scientific interest that it would generate. Therefore, this study presents the epidemiological evidence of childhood cancer risk on exposure to ELF-MFs, which implies that we can confirm the risk of childhood leukemia among pediatric cancers followed exposure to ELF-MFs, which is associated with a higher risk than what was previously known.

### Limitations

Although we identified three cohort studies that met the study eligibility criteria, these studies were not able to include in the meta-analysis. In the cohort study, the incident ratio of childhood leukemia was 1.6 and any childhood cancer was 1.5 in the group exposed to 0.2-μT, however, it was not statistically significant [38]. In the other two studies, the hazard ratio was 1.6 in the group exposed to 0.2-μT [50] and 1.9 in the group exposed to 0.3-μT [57], however, it was also not statistically significant. As the WHO recommends the conducting of cohort studies on EMF health effects, and several large-scale cohort studies are currently ongoing [62], we hope to derive an integrated value of cohort results through a systematic review in the future.

The ELF-MFs measurement variation was not considered in our meta-analysis. Although some studies used a long-term measurement, others used a spot measurement; however, no distinction has been made in our meta-analysis because of the limited number of studies. Nonetheless, our pooled results showed a non-significant heterogeneity among studies.

Another limitation is that all studies included in our study were case-control studies, which reduces the strength of the obtained results because case–control studies are subject to selection bias and other methodological problems. However, all studies that were included in this meta-analysis were the matched case-control studies except one, which had the case and control groups from the same registry. Therefore, it can be seen that the bias shown in case-control studies has diminished.

## Conclusions

In this large pooled analysis of more than 36,000 children diagnosed with childhood leukemia, statistically significant associations were observed between exposure to ELF-MF and childhood leukemia. Furthermore, the intensity of the association between exposure to ELF-MFs and childhood leukemia was high, as indicated by the dose–response effect.

The risk of ELF-MFs, which have been classified as a possibly carcinogenic (Group 2B) factor based on limited evidence in humans, can be ascertained through precise evidence from the integrated results of this study.

## Supporting information

S1 Table Search strategy in databases. Search strategies and search results for each database (Pubmed, Embase, Web of Science).
(DOCX)

S1 Checklist (DOC)

## References

[1] Staebler P. Human exposure to electromagnetic fields: from extremely low frequency (ELF) to radiofrequancy. London, UK: John Wiley & Sons; 2017.

[2] Sherrard RM, Morellini N, Jourdan N, El-Esawi M, Arthaut LD, Niessner C, et al. Low-intensity electromagnetic fields induce human cryptochrome to modulate intracellular reactive oxygen species. PLoS Biol. 2018;16(10):e2006229. Epub 2018/10/03. 10.1371/journal.pbio.2006229 30278045 PMC6168118

[3] Havas M. When theory and observation collide: Can non-ionizing radiation cause cancer? Environ Pollut. 2017;221:501–5. Epub 2016/12/03. 10.1016/j.envpol.2016.10.018 . 27903411

[4] Kheifets L, Ahlbom A, Crespi CM, Feychting M, Johansen C, Monroe J, et al. A pooled analysis of extremely low-frequency magnetic fields and childhood brain tumors. Am J Epidemiol. 2010;172(7):752–61. Epub 2010/08/11. 10.1093/aje/kwq181 20696650 PMC2984256

[5] Kheifets L, Ahlbom A, Crespi CM, Draper G, Hagihara J, Lowenthal RM, et al. Pooled analysis of recent studies on magnetic fields and childhood leukaemia. Br J Cancer. 2010;103(7):1128–35. Epub 2010/09/30. 10.1038/sj.bjc.6605838 20877339 PMC2965855

[6] Schüz J, Dasenbrock C, Ravazzani P, Röösli M, Schär P, Bounds PL, et al. Extremely low-frequency magnetic fields and risk of childhood leukemia: A risk assessment by the ARIMMORA consortium. Bioelectromagnetics. 2016;37(3):183–9. Epub 2016/03/19. 10.1002/bem.21963 . 26991812

[7] IARC Working Group on the Evaluation of Carcinogenic Risks to Humans. Non-ionizing radiation, Part 1: static and extremely low-frequency (ELF) electric and magnetic fields. IARC Monogr Eval Carcinog Risks Hum. 2002;80:1–395. Epub 2002/06/20. 12071196 PMC5098132

[8] World Health Organization, International Labour Organisation, International Commission on Non-Ionizing Radiation Protection. Extremely low frequency fields. Geneva: World Health Organization; 2007.

[9] Linet MS, Hatch EE, Kleinerman RA, Robison LL, Kaune WT, Friedman DR, et al. Residential exposure to magnetic fields and acute lymphoblastic leukemia in children. N Engl J Med. 1997;337(1):1–7. 10.1056/NEJM199707033370101 9203424

[10] Dockerty JD, Elwood JM, Skegg DC, Herbison GP. Electromagnetic field exposures and childhood cancers in New Zealand. Cancer Causes Control. 1998;9(3):299–309. 10.1023/a:1008825220759 9684710

[11] Bianchi N, Crosignani P, Rovelli A, Tittarelli A, Carnelli CA, Rossitto F, et al. Overhead electricity power lines and childhood leukemia: a registry-based, case-control study. Tumori. 2000;86(3):195–8. 10939597 10.1177/030089160008600303

[12] Salvan A, Ranucci A, Lagorio S, Magnani C. Childhood Leukemia and 50 Hz Magnetic Fields: Findings from the Italian SETIL Case-Control Study. Int J Environ Res Public Health. 2015;12(2):2184–204. 10.3390/ijerph120202184 25689995 PMC4344719

[13] World Health Organization. 2007 WHO Research Agenda for Extremely Low Frequency Fields Geneva: World Health Organization; 2007. Available from: https://www.who.int/publications/m/item/2007-who-research-agenda-for-extremely-low-frequency-fields.

[14] Ahlbom A, Day N, Feychting M, Roman E, Skinner J, Dockerty J, et al. A pooled analysis of magnetic fields and childhood leukaemia. Br J Cancer. 2000;83(5):692–8. Epub 2000/08/17. 10.1054/bjoc.2000.1376 10944614 PMC2363518

[15] Kroll ME, Swanson J, Vincent TJ, Draper GJ. Childhood cancer and magnetic fields from high-voltage power lines in England and Wales: a case-control study. Br J Cancer. 2010;103(7):1122–7. 10.1038/sj.bjc.6605795 20877338 PMC2965853

[16] Malagoli C, Fabbi S, Teggi S, Calzari M, Poli M, Ballotti E, et al. Risk of hematological malignancies associated with magnetic fields exposure from power lines: a case-control study in two municipalities of northern Italy. Environ Health. 2010;9(1):16–1—8. 10.1186/1476-069X-9-16 20353586 PMC2856548

[17] Saito TN, Hiroshi Kubo, Osami Yamamoto, Seiichiro Yamaguchi, Naohito Akiba, Suminori Honda, et al. Power-frequency magnetic fields and childhood brain tumors: a case-control study in Japan. Journal Of Epidemiology. 2010;20(1):54–61. 10.2188/jea.je20081017 . 19915304 PMC3900780

[18] Does M, Scelo G, Metayer C, Selvin S, Kavet R, Buffler P. Exposure to electrical contact currents and the risk of childhood leukemia. Radiat Res. 2011;175(3):390–6. 10.1667/RR2357.1 21388283 PMC3087620

[19] Wünsch Filho V, Pelissari DM, Barbieri FE, Sant Anna L, de Oliveira CT, de Mata JF, et al. Exposure to magnetic fields and childhood acute lymphocytic leukemia in Sao Paulo, Brazil. Cancer Epidemiol. 2011;35(6):534–9. 10.1016/j.canep.2011.05.008 21840286

[20] Jirik V, Pekarek L, Janout V, Tomaskova H. Association between Childhood Leukaemia and Exposure to Power-frequency Magnetic Fields in Middle Europe. Biomed Environ Sci. 2012;25(5):597–601. 10.3967/0895-3988.2012.05.015 23122319

[21] Ba Hakim AS, Abd Rahman NB, Mokhtar MZ, Said IB, Hussain H. ELF-EMF correlation study on distance from overhead transmission lines and acute leukemia among children in Klang Valley, Malaysia. IEEE Conference on Biomedical Engineering and Sciences (IECBES), 2014. 1 ed: IEEE; 2014. p. 710–4.

[22] Bunch KJ, Swanson J, Vincent TJ, Murphy MF. Magnetic fields and childhood cancer: an epidemiological investigation of the effects of high-voltage underground cables. J Radiol Prot. 2015;35(3):695–705. 10.1088/0952-4746/35/3/695 26344172

[23] Pedersen C, Johansen C, Schüz J, Olsen JH, Raaschou-Nielsen O. Residential exposure to extremely low-frequency magnetic fields and risk of childhood leukaemia, CNS tumour and lymphoma in Denmark. Br J Cancer. 2015;113(9):1370–4. 10.1038/bjc.2015.365 26484412 PMC4815792

[24] Kheifets L, Crespi CM, Hooper C, Cockburn M, Amoon AT, Vergara XPJCC, et al. Residential magnetic fields exposure and childhood leukemia: a population-based case–control study in California. 2017;28(10):1117–23. 10.1007/s10552-017-0951-6 28900736 PMC5706765

[25] Crespi CM, Swanson J, Vergara XP, Kheifets LJEr. Childhood leukemia risk in the California Power Line Study: Magnetic fields versus distance from power lines. 2019;171:530–5. 10.1016/j.envres.2019.01.022 PMC6392457 30743245

[26] Wertheimer N, Leeper E. Electrical wiring configurations and childhood cancer. Am J Epidemiol. 1979;109(3):273–84. Epub 1979/03/01. 10.1093/oxfordjournals.aje.a112681 . 453167

[27] Higgins JPT. Cochrane handbook for systematic reviews of interventions. 2nd ed. Hoboken, NJ: Wiley-Blackwell; 2019.

[28] Moher D, Shamseer L, Clarke M, Ghersi D, Liberati A, Petticrew M, et al. Preferred reporting items for systematic review and meta-analysis protocols (PRISMA-P) 2015 statement. Syst Rev. 2015;4(1):1. Epub 2015/01/03. 10.1186/2046-4053-4-1 25554246 PMC4320440

[29] Marcano Belisario JS, Tudor Car L, Reeves TJA, Gunn LH, Car J. Search strategies to identify observational studies in MEDLINE and EMBASE. Cochrane Database Syst Rev. 2013;(12). 10.1002/14651858.MR000041 MR000041. PMC8103566 30860595

[30] Wells GA. The Newcastle-Ottawa Scale (NOS) for assessing the quality of nonrandomized studies in meta-analyses. 2015.

[31] Higgins JP, Thompson SG. Quantifying heterogeneity in a meta-analysis. Stat Med. 2002;21(11):1539–58. Epub 2002/07/12. 10.1002/sim.1186 . 12111919

[32] Higgins JPT, Thompson SG, Deeks JJ, Altman DG. Measuring inconsistency in meta-analyses. Br Med J. 2003;327(7414):557–60. 10.1136/bmj.327.7414.557 12958120 PMC192859

[33] Savitz DA, Wachtel H, Barnes FA, John EM, Tvrdik JG. Case-control study of childhood cancer and exposure to 60-Hz magnetic fields. Am J Epidemiol. 1988;128(1):21–38. 10.1093/oxfordjournals.aje.a114943 3164167

[34] Myers A, Clayden AD, Cartwright RA, Cartwright SC. Childhood cancer and overhead powerlines: a case-control study. Br J Cancer. 1990;62(6):1008–14. 10.1038/bjc.1990.428 2257204 PMC1971546

[35] London SJ, Thomas DC, Bowman JD, Sobel E, Cheng TC, Peters JM. Exposure to residential electric and magnetic fields and risk of childhood leukemia. Am J Epidemiol. 1991;134(9):923–37. 10.1093/oxfordjournals.aje.a116176 1843457

[36] Feychting M, Ahlbom A. Magnetic fields and cancer in children residing near Swedish high-voltage power lines. Am J Epidemiol. 1993;138(7):467–81. 10.1093/oxfordjournals.aje.a116881 8213751

[37] Olsen JH, Nielsen A, Schulgen G. Residence near high voltage facilities and risk of cancer in children. BMJ. 1993;307(6909):891–5. Epub 1993/10/09. 10.1136/bmj.307.6909.891 8241850 PMC1679052

[38] Verkasalo PK, Pukkala E, Hongisto MY, Valjus JE, Jarvinen PJ, Heikkila KV, et al. Risk of cancer in Finnish children living close to power lines. BMJ. 1993;307(6909):895–9. 10.1136/bmj.307.6909.895 8241851 PMC1679080

[39] Preston-Martin S, Gurney JG, Pogoda JM, Holly EA, Mueller BA. Brain tumor risk in children in relation to use of electric blankets and water bed heaters. Results from the United States West Coast Childhood Brain Tumor Study. Am J Epidemiol. 1996;143(11):1116–22. Epub 1996/06/01. 10.1093/oxfordjournals.aje.a008688 . 8633600

[40] Michaelis J, Schüz J, Meinert R, Menger M, Grigat JP, Kaatsch P, et al. Childhood leukemia and electromagnetic fields: results of a population-based case-control study in Germany. Cancer Causes Control. 1997;8(2):167–74. 10.1023/a:1018464012055 9134240

[41] Tynes T, Haldorsen T. Electromagnetic fields and cancer in children residing near Norwegian high-voltage power lines. Am J Epidemiol. 1997;145(3):219–26. 10.1093/oxfordjournals.aje.a009094 9012594

[42] Green LM, Miller AB, Villeneuve PJ, Agnew DA, Greenberg ML, Li J, et al. A case-control study of childhood leukemia in southern Ontario, Canada, and exposure to magnetic fields in residences. Int J Cancer. 1999;82(2):161–70. Epub 1999/07/02. 10.1002/(sici)1097-0215(19990719)82:2&lt;161::aid-ijc2&gt;3.0.co;2-x . 10389746

[43] McBride ML, Gallagher RP, Theriault G, Armstrong BG, Tamaro S, Spinelli JJ, et al. Power-frequency electric and magnetic fields and risk of childhood leukemia in Canada. Am J Epidemiol. 1999;149(9):831–42. 10.1093/oxfordjournals.aje.a009899 10221320

[44] Kleinerman RA, Kaune WT, Hatch EE, Wacholder S, Linet MS, Robison LL, et al. Are children living near high-voltage power lines at increased risk of acute lymphoblastic leukemia? Am J Epidemiol. 2000;151(5):512–5. 10.1093/oxfordjournals.aje.a010237 10707920

[45] UK Childhood Cancer Study Investigators. Exposure to power-frequency magnetic fields and the risk of childhood cancer. UK Childhood Cancer Study Investigators. Lancet. 1999;354(9194):1925–31. Epub 2000/01/06. . 10622294

[46] Schüz J, Grigat JP, Brinkmann K, Michaelis J. Residential magnetic fields as a risk factor for childhood acute leukaemia: results from a German population-based case-control study. Int J Cancer. 2001;91(5):728–35. 10.1002/1097-0215(200002)9999:9999&lt;::aid-ijc1097&gt;3.0.co;2-d 11267988

[47] Foliart DE, Mezei G, Iriye R, Silva JM, Ebi KL, Kheifets L, et al. Magnetic field exposure and prognostic factors in childhood leukemia. Bioelectromagnetics. 2007;28(1):69–71. 10.1002/bem.20269 16988997

[48] Kabuto M, Nitta H, Yamamoto S, Yamaguchi N, Akiba S, Honda Y, et al. Childhood leukemia and magnetic fields in Japan: a case-control study of childhood leukemia and residential power-frequency magnetic fields in Japan. Int J Cancer. 2006;119(3):643–50. 10.1002/ijc.21374 16496405

[49] Feizi AA, Arabi MA. Acute childhood leukemias and exposure to magnetic fields generated by high voltage overhead power lines—a risk factor in Iran. Asian Pac J Cancer Prev. 2007;8(1):69–72. 17477775

[50] Svendsen AL, Weihkopf T, Kaatsch P, Schüz J. Exposure to magnetic fields and survival after diagnosis of childhood leukemia: a German cohort study. Cancer Epidemiol Biomarkers Prev. 2007;16(6):1167–71. 10.1158/1055-9965.EPI-06-0887 17548680

[51] Malagoli C, Fabbi S, Teggi S, Calzari M, Poli M, Ballotti E, et al. Risk of hematological malignancies associated with magnetic fields exposure from power lines: a case-control study in two municipalities of northern Italy. Environ Health. 2010;9:16. Epub 2010/04/01. 10.1186/1476-069X-9-16 20353586 PMC2856548

[52] Saito T, Nitta H, Kubo O, Yamamoto S, Yamaguchi N, Akiba S, et al. Power-frequency magnetic fields and childhood brain tumors: a case-control study in Japan. J Epidemiol. 2010;20(1):54–61. Epub 2009/11/17. 10.2188/jea.je20081017 19915304 PMC3900780

[53] Wünsch-Filho V, Pelissari DM, Barbieri FE, Sant’Anna L, de Oliveira CT, de Mata JF, et al. Exposure to magnetic fields and childhood acute lymphocytic leukemia in São Paulo, Brazil. Cancer Epidemiol. 2011;35(6):534–9. Epub 2011/08/16. 10.1016/j.canep.2011.05.008 . 21840286

[54] Hakim ASB, Rahman NBA, Mokhtar MZ, Said IB, Hussain H. ELF—EMF correlation study on distance from Overhead Transmission Lines and acute leukemia among children in klang Valley, Malaysia. 2014 IEEE Conference on Biomedical Engineering and Sciences (IECBES); 8–10 Dec. 2014: Institute of Electrical and Electronics Engineers Inc.; 2014. p. 710–4.

[55] Kheifets L, Crespi CM, Hooper C, Cockburn M, Amoon AT, Vergara XP. Residential magnetic fields exposure and childhood leukemia: a population-based case-control study in California. Cancer Causes Control. 2017;28(10):1117–23. Epub 2017/09/14. 10.1007/s10552-017-0951-6 28900736 PMC5706765

[56] Crespi CM, Swanson J, Vergara XP, Kheifets L. Childhood leukemia risk in the California Power Line Study: Magnetic fields versus distance from power lines. Environ Res. 2019;171:530–5. Epub 2019/02/12. 10.1016/j.envres.2019.01.022 30743245 PMC6392457

[57] Foliart DE, Pollock BH, Mezei G, Iriye R, Silva JM, Ebi KL, et al. Magnetic field exposure and long-term survival among children with leukaemia. Br J Cancer. 2006;94(1):161–4. 10.1038/sj.bjc.6602916 16404370 PMC2361064

[58] Coghill RW, Steward J, Philips A. Extra low frequency electric and magnetic fields in the bedplace of children diagnosed with leukaemia: a case-control study. Eur J Cancer Prev. 1996;5(3):153–8. 10.1097/00008469-199606000-00002 8818603

[59] Feychting MS, G.:Olsen J. H.:Ahlbom A. Magnetic fields and childhood cancer—A pooled analysis of two Scandinavian studies. European Journal of Cancer Part A: General Topics. 1995;31(12):2035–9. 10.1016/0959-8049(95)00472-6 8562161

[60] Turner RM, Davey J, Clarke MJ, Thompson SG, Higgins JP. Predicting the extent of heterogeneity in meta-analysis, using empirical data from the Cochrane Database of Systematic Reviews. Int J Epidemiol. 2012;41(3):818–27. Epub 2012/03/31. 10.1093/ije/dys041 22461129 PMC3396310

[61] Kandel S, Swanson J, Kheifets L. Health-Economics Analyses Applied to ELF Electric and Magnetic Fields. Risk Anal. 2016;36(6):1277–86. Epub 2016/01/23. 10.1111/risa.12551 . 26800316

[62] World Health Organization. International EMF Project Geneva: World Health Organization; 2018. Available from: https://www.who.int/peh-emf/project/en/.

