# Supplementary material for: Exposure to extremely low-frequency magnetic fields and childhood cancer: A systematic review and meta-analysis
Source: PLoS One. 2021 May 14;16(5):e0251628. doi: 10.1371/journal.pone.0251628 (PMC8121331; doi:10.1371/journal.pone.0251628)
Supplement: S1 Table — Search strategies and search results for each database (Pubmed, Embase, Web of Science). (DOCX) [file pone.0251628.s001.docx]

**S1 Table.** Search strategy in databases

|  | **Searches (Pubmed)** | **Results** |
| --- | --- | --- |
| #1 | "electromagnetic fields"[MeSH Terms] OR ("electromagnetic"[All Fields] AND "fields"[All Fields]) OR "electromagnetic fields"[All Fields] OR ("electromagnetic"[All Fields] AND "field"[All Fields]) OR "electromagnetic field"[All Fields] | 26,120 |
| #2 | "non ionising radiation"[All Fields] OR "radiation, nonionizing"[MeSH Terms] OR ("radiation"[All Fields] AND "nonionizing"[All Fields]) OR "nonionizing radiation"[All Fields] OR ("non"[All Fields] AND "ionizing"[All Fields] AND "radiation"[All Fields]) OR "non ionizing radiation"[All Fields] | 25,506 |
| #3 | "child"[MeSH Terms] OR "child"[All Fields] | 2,018,855 |
| #4 | "adolescent"[MeSH Terms] OR "adolescent"[All Fields] | 1,965,426 |
| #5 | "epidemiologic studies"[MeSH Terms] OR ("epidemiologic"[All Fields] AND "studies"[All Fields]) OR "epidemiologic studies"[All Fields] | 2,409,681 |
| #6 | "case-control studies"[MeSH Terms] OR ("case-control"[All Fields] AND "studies"[All Fields]) OR "case-control studies"[All Fields] OR ("case"[All Fields] AND "control"[All Fields] AND "study"[All Fields]) OR "case control study"[All Fields] | 1,175,065 |
| #7 | "cohort studies"[MeSH Terms] OR ("cohort"[All Fields] AND "studies"[All Fields]) OR "cohort studies"[All Fields] OR "cohort"[All Fields] | 2,191,530 |
| #8 | "observational study"[Publication Type] OR "observational studies as topic"[MeSH Terms] OR "observational study"[All Fields] | 122,092 |
| #9 | #1 OR #2 | 291,208 |
| #10 | #3 OR #4 | 3,192,285 |
| #11 | #5 OR #6 OR #7 OR #8 | 2,741,106 |
| #12 | #9 AND #10 | 13,905 |
| #13 | #11 AND #12 | 2,635 |
|  | **Searches (Embase)** | **Results** |
| #1 | 'electromagnetic radiation'/exp | 518,410 |
| #2 | non AND ionizing AND radiation | 7,576 |
| #3 | electromagnetic AND field | 12,200 |
| #4 | 'child'/exp | 2,791,765 |
| #5 | 'adolescent'/exp | 1,582,924 |
| #6 | 'case control study'/exp | 191,315 |
| #7 | 'cohort analysis'/exp | 569,085 |
| #8 | epidemiological AND study | 170,069 |
| #9 | 'observational study'/exp | 187,601 |
| #10 | #1 OR #2 OR #3 | 529,031 |
| #11 | #4 OR #5 | 3,491,680 |
| #12 | #6 OR #7 OR #8 OR #9 | 978,596 |
| #13 | #10 AND #11 | 23,830 |
| #14 | #12 AND #13 | 1,012 |
|  | **Searches (Web of Science)** | **Results** |
| #1 | Electromagnetic field | 95,550 |
| #2 | non ionizing radiation | 7,016 |
| #3 | child | 1,548,902 |
| #4 | adolescent | 394,812 |
| #5 | case control | 569,804 |
| #6 | cohort | 640,212 |
| #7 | observational study) | 175,863 |
| #8 | epidemiological study) | 100,230 |
| #9 | #1 OR #2 | 95,635 |
| #10 | #3 OR #4 | 1,702,850 |
| #11 | #5 OR #6 OR #7 OR #8 | 1,310,911 |
| #12 | #9 AND #10 | 960 |
| #13 | #11 AND #12 | 265 |
